# Impact of Armed Conflicts on Public Health Infrastructure and Services in Oromia, Ethiopia

**DOI:** 10.7759/cureus.40653

**Published:** 2023-06-19

**Authors:** Girma Gutema, Mirgissa Kaba, Zewdie Birhanu, Jilcha Diribi, Ibrahim Elemo

**Affiliations:** 1 Pharmacology, Rift Valley University, Adama, ETH; 2 Community Health, School of Public Health, College of Health Sciences, Addis Ababa University, Addis Ababa, ETH; 3 Epidemiology and Public Health, School of Public Health, Jimma University, Jimma, ETH; 4 General Practice, Oromia Physicians Association, Addis Ababa, ETH; 5 Hospital Medicine, University of Minnesota Medical Center, Minnesota, USA

**Keywords:** ethiopia, oromia, health infrastructure, public health services, impacts, armed conflicts

## Abstract

Background

Oromia is the largest national regional state in the Ethiopian federation. It covers over a third of the country’s landmass. In terms of sheer geography, Oromia is about the size of the sovereign European state of Germany. Demographically, Oromia closely matches with Poland among other European countries. Since early 2019, there are actively ongoing armed conflicts in Oromia damaging the public health infrastructure and hampering the provision of healthcare services.

Objective

The objective of this study is to assess and document the impacts of armed conflicts in Oromia on the public health infrastructure.

Method

The study is a quantitative review of administrative records and reports employing a qualitative analytical prism.

Results

Oromia has 22 administrative zones of which 11 (50%) host 142 sites sheltering about 1.5 million Internally Displaced Persons (IDPs). A total of 1072 public healthcare facilities sustained attacks in areas of armed conflicts across Oromia. Among the 159 motor vehicles attacked (ambulances, district health office cars and motorbikes), 44% were ambulances. Only for the first two weeks of January 2023, 25,580 Severe Acute Malnutrition (SAM) cases were reported by healthcare facilities from the areas affected by armed conflicts in Oromia. In these areas, 11,740 patients with malnutrition were enrolled into the Outpatient Therapeutic Program (OTP), 1050 were put on subcutaneous infusion (SC) and seven died due to SAM only in the first two weeks of January 2023. Severe droughts that happened for five consecutive rainy seasons over the last three years have hit hard 10 administrative zones in Oromia, thereby compounding the impacts of the armed conflicts.

Conclusions

Armed conflicts are damaging the public health infrastructure and hampering healthcare provisions in Oromia. Such conflicts are evicting people from their residential places thereby forcing them to live in poorly thatched out temporary shelters with clear implication for serious health crises. When compounded with natural calamities such as climate-change-driven drought, the impacts of such conflicts on public health infrastructure and the resultant constraints on provision of vital public healthcare services would be paramount. The authors recommend for further detailed studies on the sustained impacts that these armed conflicts can possibly bring on the provision of vital public health services in Oromia.

## Introduction

Oromia is the largest of the 11 national regional states in the Ethiopian multinational federation [1], covering over a third of the country's 1.1 million square kilometers landmass [2]. In terms of shear geography, Oromia is about the size of the sovereign European state of Germany [3, 4]. Demographically, Oromia closely matches with Poland among other European countries with about 40 million inhabitants [5, 6].

The 2018 estimate of Ethiopia’s Statistics Agency shows that among Oromia’s total population, about 85% reside in rural areas [7]. The regional state is divided into 22 administrative zones (Godinaalee, in Afaan Oromoo, the language of the Oromo people), 20 urban centers and 333 local districts, per the official report of the Oromia Health Bureau entitled “Karoora Tiraansfoormeeshinii Seektara Fayyaa Lammaffaa (HSTP-II)” [8]. The healthcare system in Oromia is organized into three tiers all accountable to the Oromia Health Bureau [9].

Since early 2019, there are actively ongoing armed conflicts in Oromia regional state [10] which might have severely damaged the public health infrastructure and services in Oromia. The conflicts pit the federal government of Ethiopia with the Oromo Liberation Army (OLA), an armed splinter group of the old and influential political party in Oromia known as the Oromo Liberation Front (OLF) [11].

Generally, there is an absolute scarcity of literature on the impacts of armed conflicts on public health infrastructure and services in other Horn of African countries. This reality limits the possibility to further expand and enrich this section of our article with more scientific resources. But particularly and more importantly for this research, nothing, literally nothing, is known on the extent to which these armed conflicts have been damaging the public health infrastructure and provision of healthcare services in Oromia.

Documenting the impact of the armed conflicts on the public health services and infrastructure in Oromia is imperative. This would not only help to give a better understanding on the health situation of the region in conflict thorn areas, but also capture grounded data on the collateral and/or non-collateral damages sustained by healthcare infrastructure due to armed conflicts.

Such data can also be vital inputs for policymakers and humanitarian aid agencies who are in a position to redress the damages or to mitigate the impacts of armed conflicts on the provision of essential public healthcare services.

The aim of this study (earlier draft manuscript previously posted to a pre-print server Research Square on April 27, 2023) is therefore to assess and document the impacts of the raging armed conflicts in Oromia on the public health infrastructure and their practical repercussions on healthcare service provision to the communities in need.

## Materials and methods

Study type and data sources

The study is based on a retrospective quantitative evaluation of administrative records and emergency reports publicly available at the Oromia Health Bureau. It employs an analytical prism in interpreting the official data.

As the armed conflicts expand across Oromia both in scope and severity, the ever-increasing number of Internally Displaced Peoples (IDPs) and the damages sustained by healthcare facilities as well as health emergency transportation systems necessitated taking urgent measures, including the need to undertake tailored assessment of the ground realities on these parameters. Accordingly, the Oromia Health Bureau organized a comprehensive study in which data were collected from all the conflict-affected administrative zones in the regional state. The assessment documented the geographic distribution of the conflicts in Oromia, the number of conflict-ravaged woredas (localities), the number of IDPs, including household units, as well as the number of communal sites where these IDPs were temporarily settled.

Also documented were the number of healthcare facilities (hospitals, health centers and health posts) and emergency health transportation systems (ambulances, motorbikes and other vehicles) which sustained attacks in each of the administrative zones affected by the conflicts in Oromia. Moreover, another assessment, undertaken concurrently, documented the distribution of administrative zones severely affected by climate-change-driven drought as compounding the problems caused by armed conflicts. These studies also documented some of the emergency healthcare responses provided at IDP sites and drought-afflicted areas including screening of malnutrition, distribution of emergency food items and medicines and screening of chronic diseases like TB and HIV/AIDS, among others.

The data in this article are based on extracts from these assessments, as systematically curated to find out the impacts of the raging armed conflicts on the public health infrastructure and services in Oromia. Reports from Non-Governmental Organizations (NGOs) operating remotely or within the conflict-affected settings were also consulted. Besides, credible media reports pertinent to the study theme were scanned for brief reviews.

Data management and analysis

Curated data were organized, entered and analyzed using SPSS Statistics, Version 26 (IBM Corp., Armonk, NY, USA). Descriptive statistics were used to summarize the basic feature of data on the study theme. Results were compiled into the following major categories to capture the impacts of the conflict, from 2020 to 2023: 1) Conflict-driven Internally Displaced Peoples (IDPs), 2) damages on healthcare facilities, 3) damages on ambulances and other vehicles used by healthcare facilities for logistics, 4) conflict-driven malnutrition and, 5) drought further compounding impacts of the conflict in Oromia. The resultant data sets were presented in texts and figures.

## Results

Distributions of IDPs by administrative zones

**Figure 1:**
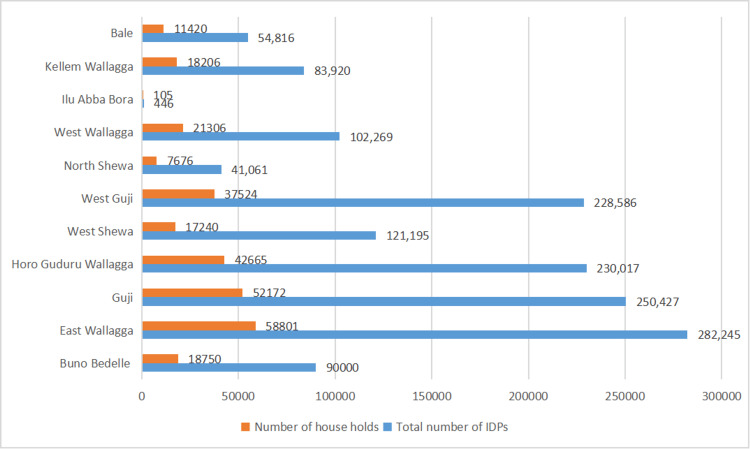
Distribution of conflict-driven IDPs and the number of their households in 11 administrative zones in Oromia, as of January 2023. IDPs: Internally Displaced Persons

Oromia has 22 administrative zones of which 11 (50%) host a total of 142 sites sheltering about 1.5 million Internally Displaced Persons (IDPs), all caused by conflicts. Figure 1 shows the distribution of IDPs and the total number of their households in each of the 11 administrative zones across Oromia, as of the first week of January 2023.

Damages caused to healthcare facilities

**Figure 2:**
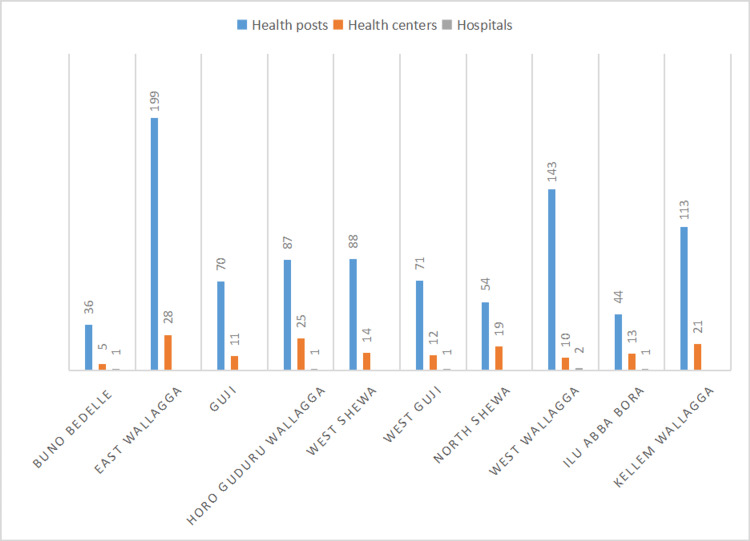
Distribution of health posts, health centers and hospitals which were damaged, looted, completely destroyed and/or burned in the 11 administrative zones affected by the conflict across Oromia, as of the first week of January 2023.

A total of 1072 public healthcare facilities were reported to have been damaged, looted, completely destroyed and/or burned across Oromia in the areas where the armed conflicts are raging. Figure 2 depicts the distribution of health posts, health centers and hospitals which were damaged, looted, completely destroyed and/or burned in the 11 administrative zones across Oromia where active conflicts were underway.

**Figure 3:**
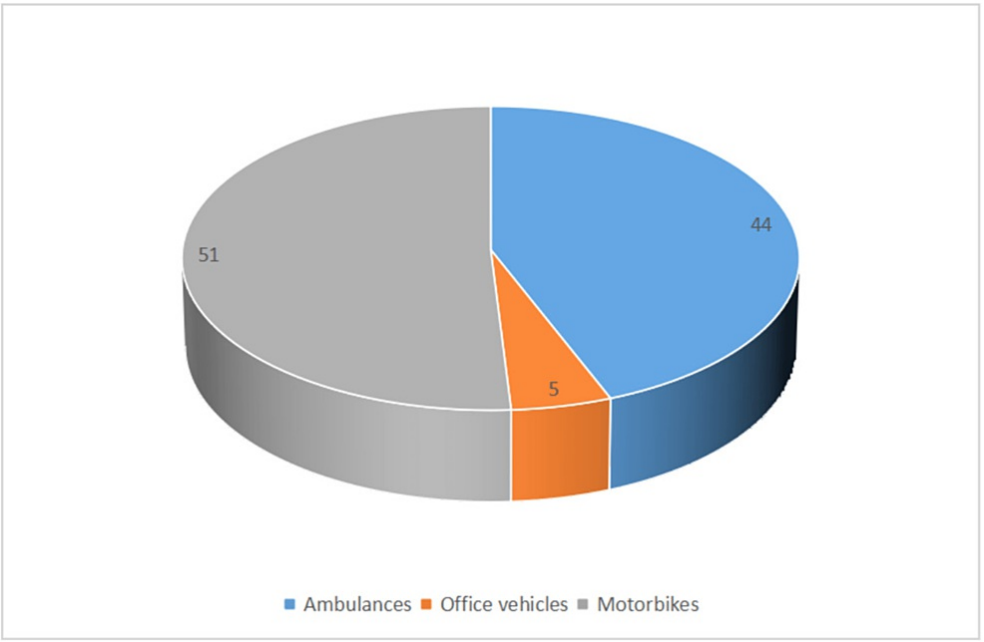
Percentage of motor vehicles (ambulances, district health office cars, motorbikes) used for providing regular health emergency services in the areas severely affected by armed conflicts in Oromia, as officially reported to be damaged, looted and/or destroyed due to the conflicts, as of January 2023.

Among the 159 motor vehicles (ambulances, district health office cars for logistics, motorbikes) used for providing regular health emergency services in the areas severely affected by the ongoing armed conflicts in Oromia, as officially been reported to be damaged, looted and/or destroyed due to these armed conflicts, ambulances accounted for 44% (Figure 3).

Severe and acute malnutrition (SAM)

Data from the conflict-affected areas show that the burden of Severe and Acute Malnutrition (SAM) has increased substantially. Only for the first two weeks of January 2023, 25,580 SAM cases were reported by healthcare facilities from within the areas affected by the ongoing conflicts in Oromia.

In these areas, 11,740 patients with malnutrition were enrolled into the Outpatient Therapeutic Program (OTP), 1050 were put on subcutaneous infusion (SC) and seven died due to SAM only in the first two weeks of January 2023 in Oromia.

**Figure 4:**
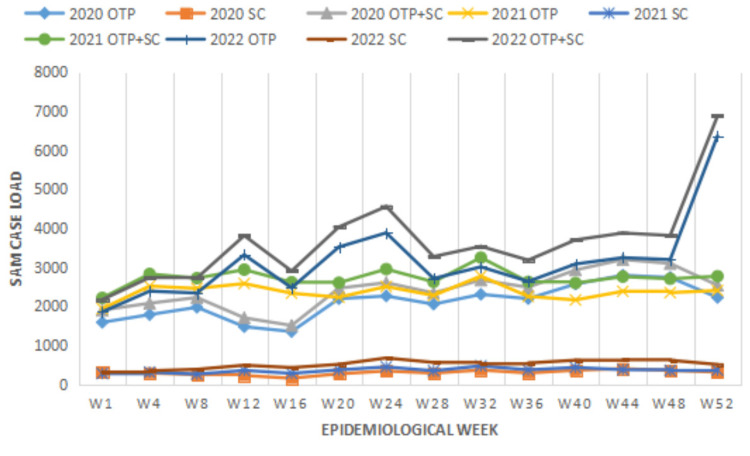
Epidemiological data trends for OTP and SC cases sampled every four weeks from the first to the last weeks for the years 2020 to 2022 in Oromia. OTP: Outpatient Therapeutic Program; SC: Subcutaneous Infusion

Figure 4 shows comparative epidemiological data trends for OTP and SC cases as taken every four weeks across all healthcare facilities from the first to the last weeks for the years 2020 to 2022 in Oromia.

Drought compounding impacts of conflicts in Oromia

**Figure 5:**
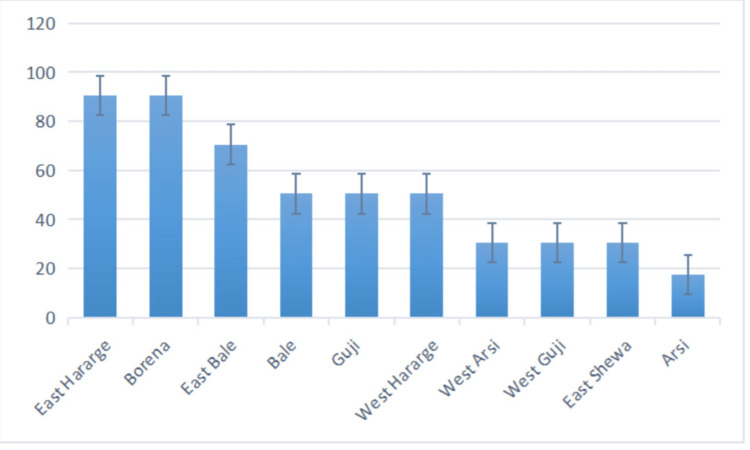
Percentage distribution of drought-affected districts in the administrative zones of Oromia, by January 2023.

Severe droughts that happened for five consecutive rainy seasons over the last three years have hit hard 10 administrative zones across the southern, eastern and south-eastern areas of Oromia, thereby effectively compounding the impacts of the armed conflicts in these areas. Figure 5 below shows the 10 administrative zones in Oromia which are affected by problems of severe environmental drought driven by climate change, as officially reported by January 2023.

## Discussion

The human and material cost of these armed conflicts in Oromia still remain unknown for many reasons of which the two most important ones being: 1) most parts of the large state of Oromia where the war rages have not been accessible for the media and academic researchers since the middle of 2019, 2) Since November 2020, the armed conflicts in Oromia have essentially been overshadowed by other devastating armed conflicts in Tigray [12] which pitted Ethiopia’s federal government with Tigray regional state authorities, as ruled by the Tigray Peoples’ Liberation Front or TPLF for short.

The later conflicts now appear to have ended after the African Union-led negotiations on the permanent ceasefire agreement signed by both parties to the conflict in Pretoria, South Africa, in November 2022 [13].

Pertaining to the evolution of the conflicts in Oromia, when the OLF agreed to return to Ethiopia from its military base in Eritrea to take part in a peaceful political struggle by abandoning armed struggle based on an "obscured political deal" it stroke with the federal government of Ethiopia, some units of its military wing known as Oromo Liberation Army (OLA) in Western and Southern Oromia areas rejected the idea of laying down their arms, claiming that the negotiations made between the OLF and Ethiopia’s federal government ignored the involvement of fighters on the ground in these parts of Oromia. The splinter group then effectively declared its disassociation with the OLF and vowed to continue with the armed struggle [14].

We argue that the armed conflicts now covering over half of Oromia effectively radiated to other parts of the state from the strongholds of OLA in these western and southern hinterlands since 2019, and hence the reason why these areas in Oromia are the most affected with regard to the conflict-driven IDP numbers to this date, for instance.

As is the case in other members of the Ethiopian multinational federation [15], the healthcare system in Oromia is organized into three tiers: 1) Primary healthcare unit which includes health posts, health centers and primary hospitals, 2) General hospitals which provide secondary care, 3) Specialized hospitals which provide tertiary care. The first and the second tiers in Oromia’s healthcare system are the ones very close to local communities and hence serve most of the populations at local levels in all the districts. These are among the most severely affected by the armed conflict in Oromia, as the results in this study showed.

East Wallagga, Guji, Horo Guduru Wallagga and West Guji are the most severely affected administrative zones in connection to armed conflict and consequent number of IDPs in Oromia. Over a quarter of a million IDPs are on the official registry at the Oromia Health Department for each of these administrative zones. The reason for this might be explained by the prevailing politics of violence in Oromia post-2018, which was hitherto peaceful and pro-democratic movement that heralded the so-called “reform process” which ultimately ushered in the era of the incumbent “prosperity party” administration in Ethiopia [10].

Given that such healthcare facilities provide essential services including infant and child immunization, maternity as well as child health services among others, it’s reasonable to assume that the damage done to these healthcare facilities due to the conflict will have long-lasting consequences on the public health conditions in Oromia. There were a total of 79 public hospitals, 1383 health centers and 6797 health posts in Oromia by 2018 [8]. If we estimate the damages done to healthcare facilities due to the war based on these official data, it can be seen that, respectively, about 8%, 12% and 14% of hospitals, health centers and health posts have been damaged, looted and/or completely destroyed due to the ongoing armed conflict in Oromia.

Ambulances and motorbikes are key in providing health emergency services in Oromia. Both of these transportation tools sustained severe damages due to the armed conflict. Of course, other vehicles owned and used by district health offices for administrative purposes have also been damaged/destroyed due to the war. Yet certainly, the damages to ambulances and motorbikes, both being the mainstay of transportation services for health emergencies in Oromia, could contribute to the major impacts that the conflict might have had on the provision of basic public health services in Oromia so far.

The other serious impact of the conflict on the public health infrastructure in Oromia is demonstrated by the sharp rise in the number of malnourished patients enrolled in OTP, SC and also those who died due to SAM. Reports of OTP and SC cases have been rising every year since 2019 when the armed conflict started to intensify in Oromia. Death records were however rare occurrences for most parts of the epidemiological weeks. Nevertheless, only for the first two weeks of 2023, seven patients were reported dead due to SAM. The fact that detection rate of all SAM cases in Oromia, Ethiopia, being so low [16] would mean a lot in understanding the real meaning of these figures.

Moreover, given that the war has been intensifying even more as these official reports were trickling in, these figures must be read as a red flag flying on the face of an imminent humanitarian disaster set to unfold in Oromia.

Looking at trend data of epidemiological weeks for SAM cases (OTP+SC) between 2020 and 2022 in Oromia gives a glaring picture of the devastating humanitarian disaster unfolding on the ground. For the first epidemiological week of the year 2020, for instance, total SAM cases (OTP+SC) were about 2000. For the last epidemiological week in 2022, however, over 6800 SAM cases (OTP+SC) were reported. This being over a three-fold increase, we argue that it shows a compounded effect of armed conflict and severe drought in Oromia.

As demonstrated in Figure 4, there were notable increases in SAM cases between 2020 and 2022 for the first epidemiological weeks, but the increasing rates did not show a sharp rise as is the case for the last weeks of 2022. All along, the highest record for SAM cases (OTP+SC) was registered for epidemiological week 52 of the year 2022. The reason for this might be because of the severe drought that inflicted the whole of the Horn of Africa region [17] which is heavily compounding the impacts of the war in Oromia. In a related point, some more devastating impacts that armed conflicts have had on the public health infrastructure, services and food insecurity in Tigray, another regional state in Ethiopia, were reported elsewhere [18].

What's more, such severe drought as driven by climate change appears to have fueled the public health crisis caused by the conflict in Oromia. In the administrative zones like Borena and East Hararge, the severe drought and all its devastating impacts have already caused famine in which over three million heads of livestock have died and the livelihoods of about one million people reduced to rubble. All these people are now in need of food aid for survival.

In the other drought-afflicted administrative zones in Oromia like East Bale, and Guji, the severe droughts are likely to turn into famine before the arrival of the main rainy season in April to June unless proactive interventions are urgently made.

The strength of this study is that it quantitatively documented the impact that the ongoing armed conflict in the Oromia state of Ethiopia is having on the public health infrastructure and services. Possible limitations of the study could include under-reporting of damages sustained by healthcare infrastructures in areas of the war zone, primarily due to the difficulty of outreach, as is the case in such situations anywhere in the world. Moreover, data on key indicators of the impact of the conflicts like the number of IDPs and malnutrition cases are only aggregate ones. This means that, for instance, they are not disaggregated enough to show distributions by age and gender across the administrative zones in Oromia.

## Conclusions

Armed conflicts are damaging the public health infrastructure in Oromia, thereby seriously constraining the provision of public health services to the general public in the regional state. Armed conflicts evict people from their safe residential places thereby forcing them to live in poorly thatched out temporary shelters. The implication of the health crisis in such cases is a logical outcome. When compounded with natural calamities such as climate-change-driven drought, the impacts and implications of armed conflicts on the public health infrastructure and the resultant constraints put on the provision of vital public healthcare services would be paramount. With such early bird pitching, the authors recommend for further detailed studies on the sustained impacts that these armed conflicts can possibly bring on the provision of vital public health services in Oromia.
